# Perceived Fatigue and Associated Psychological Factors in Patients with Myasthenia Gravis

**DOI:** 10.3390/healthcare14030342

**Published:** 2026-01-29

**Authors:** Weronika Jung-Plath, Marcelina Skrzypek-Czerko, Agata Zdun-Ryżewska, Małgorzata Bilińska, Wioletta Mędrzycka-Dąbrowska

**Affiliations:** 1 Division of Neurological and Psychiatric Nursing, Faculty of Health Sciences, Medical University of Gdańsk, Dębinki 7, 80-211 Gdańsk, Poland; weronika.jung-plath@gumed.edu.pl (W.J.-P.); marcelina.skrzypek-czerko@gumed.edu.pl (M.S.-C.); 2 Division of Quality of Life Research, Faculty of Health Sciences, Medical University of Gdańsk, Dębinki 7, 80-211 Gdańsk, Poland; 3 Department of Adult Neurology, University Clinical Center, Dębinki 7, 80-211 Gdańsk, Poland; 4 Department of Anaesthesiology Nursing & Intensive Care, Faculty of Health Sciences, Medical University of Gdansk, Dębinki 7, 80-211 Gdańsk, Poland

**Keywords:** myasthenia gravis, fatigue, perception, quality of life, psychosocial support

## Abstract

**Introduction**: Myasthenia gravis (MG) is a chronic autoimmune disorder in which fatigue represents one of the most burdensome symptoms. This multidimensional manifestation extends beyond neuromuscular fatigability and has a substantial impact on daily functioning, mental health, and quality of life. The present study aimed to evaluate the perception of fatigue in patients with MG, with particular emphasis on its interference with everyday activities and the extent to which it is understood by others. **Methods**: The study included 67 MG patients (61.2% women, mean age 53 years) treated at the Neurology Outpatient Department of the University Clinical Center in Gdańsk. Data were collected using an author-developed survey and standardized instruments: Chalder Fatigue Scale (CFQ), MG-ADL, MG-QoL15, HADS-M, Mini-COPE, and ACDS. **Results**: More than 70% of patients reported constant or frequent fatigue. Higher fatigue severity was positively associated with functional impairment (MG-ADL) and lower quality of life (MG-QoL15). More than 70% of patients reported constant or frequent fatigue. Higher fatigue severity was moderately associated with greater functional impairment and poorer quality of life. The extent to which fatigue interfered with daily life was associated with higher levels of depressive symptoms, poorer self-rated health, and less favorable disease-related perceptions (acceptance and influence). In contrast, perceiving fatigue as being better understood by others was associated with lower anxiety and depression and more favorable disease-related perceptions (acceptance, control, understanding), while it was not significantly related to fatigue severity, functional status, or quality of life. **Conclusions**: Fatigue in myasthenia gravis is a prevalent symptom, closely related to functional impairment and reduced quality of life. Different aspects of fatigue perception show distinct psychosocial correlates, highlighting the importance of considering subjective and social dimensions of fatigue alongside its severity. These findings support the relevance of psychosocial factors in the comprehensive care of patients with MG.

## 1. Introduction

Myasthenia gravis (MG) is a chronic autoimmune disease characterized by fatigue and weakness of the skeletal muscles, which worsens during exertion and subsides after rest. Symptoms may involve the ocular, bulbar, limb, and respiratory muscles, and their variable course makes both diagnosis and effective treatment difficult [1,2,3]. In recent years, increasing attention has been paid to non-motor symptoms, particularly fatigue, which, alongside muscle fatigue, is one of the most commonly reported problems associated with MG. Classic studies have shown that a distinction should be made between objective fatigability and subjective perception of fatigue, which do not always correlate with each other, confirming the multidimensional nature of this symptom. Similar observations have been confirmed in more recent studies indicating that fatigue can increase independently of muscle strength and can be associated with depressive symptoms or sleep disorders [1,3,4]. The literature emphasizes that chronic fatigue affects 60% to 80% of MG patients and significantly impacts their daily functioning, reducing their quality of life and daily activity [5,6]. Systematic reviews have shown that central fatigue is particularly common and may be associated with, for example, a more severe course of the disease, female gender, or coexisting depressive symptoms [5,6,7]. Polish studies have also confirmed the significant impact of fatigue on the quality of life in MG patients. A study by Jung-Plath et al. (2023) showed that both physical and mental fatigue significantly reduce patients’ well-being and are associated with poorer quality of life scores [8]. Similar conclusions are contained in national reports, which draw attention to the need to distinguish between muscle fatigue and a generalized feeling of exhaustion, as well as to the psychosocial consequences of fatigue in the lives of people with MG [8,9].

While fatigue has been widely recognized as one of the most disabling non-motor symptoms in myasthenia gravis, most studies have primarily focused on its severity and impact on physical functioning and quality of life [6]. Considerably less attention has been paid to the subjective perception of fatigue and to psychosocial factors, including the extent to which fatigue is understood by patients’ social environment. Understanding fatigue not only as a physical symptom but also as a subjective and socially mediated experience may provide a more comprehensive view of its role in the daily functioning and psychological well-being of patients with myasthenia gravis [10]. Therefore, further research addressing the psychosocial context of fatigue perception is warranted.

Fatigue is increasingly recognized as a multidimensional and subjective symptom that cannot be fully explained by objective measures of disease severity alone. Previous studies in myasthenia gravis have demonstrated that fatigue is common even in clinically stable patients and may persist despite adequate pharmacological treatment. Importantly, fatigue has been associated not only with physical functioning but also with emotional distress, reduced quality of life, and difficulties in social and occupational roles. These findings highlight that fatigue in myasthenia gravis should be considered within a broader biopsychosocial framework rather than solely as a motor symptom [5,6].

Despite the growing body of research on fatigue in myasthenia gravis, existing studies have focused predominantly on fatigue severity, physical functioning, and quality of life. Less attention has been paid to patients’ subjective appraisal of fatigue, particularly how fatigue is experienced as interfering with everyday life and how it is perceived and understood within the social environment. Moreover, the psychosocial correlates of fatigue perception, including emotional distress, disease-related beliefs, coping strategies, and treatment adherence, remain insufficiently explored.

Consequently, there is a lack of evidence regarding whether different aspects of fatigue perception are differentially associated with psychological and behavioral outcomes in patients with myasthenia gravis.

### Research Questions

The present study was guided by the following research questions:

1. How is fatigue perceived by patients with myasthenia gravis in terms of its interference with daily life?
2. To what extent do patients with myasthenia gravis perceive that their fatigue is understood by people in their social environment (e.g., family, caregivers, healthcare professionals)?
3. How are different aspects of fatigue perception associated with patients’ functioning, quality of life, psychological well-being, coping strategies, and adherence to treatment?

## 2. Methods

### 2.1. Study Design, Setting

The study was cross-sectional in nature. The study was conducted from December 2022 to March 2023 at the Neurology Outpatient Department of the University Clinical Center in Gdańsk. Data collection was distributed across the study period and conducted during scheduled outpatient visits, which ensured inclusion of patients attending routine clinical follow-up rather than reflecting a single time-point snapshot. Patient recruitment and data collection were carried out during regular outpatient visits, which were scheduled once a week. The project was approved by the UCK Management and received a positive opinion from the Independent Bioethics Committee for Scientific Research at the Medical University of Gdańsk, No. 769/2022. All participants gave their informed written consent to participate in the study.

### 2.2. Participants

The study included 67 patients diagnosed with myasthenia gravis (61.2% women) and was conducted at the neurology clinic for patients with myasthenia gravis attached to the Department of Adult Neurology of the University Clinical Center in Gdańsk.

Patients were recruited consecutively during routine outpatient visits. Inclusion criteria comprised a confirmed diagnosis of myasthenia gravis and the ability to provide informed consent. Exclusion criteria included lack of consent to participate in the study and patients undergoing diagnostic evaluation without confirmed myasthenia gravis.

**Figure 1:**
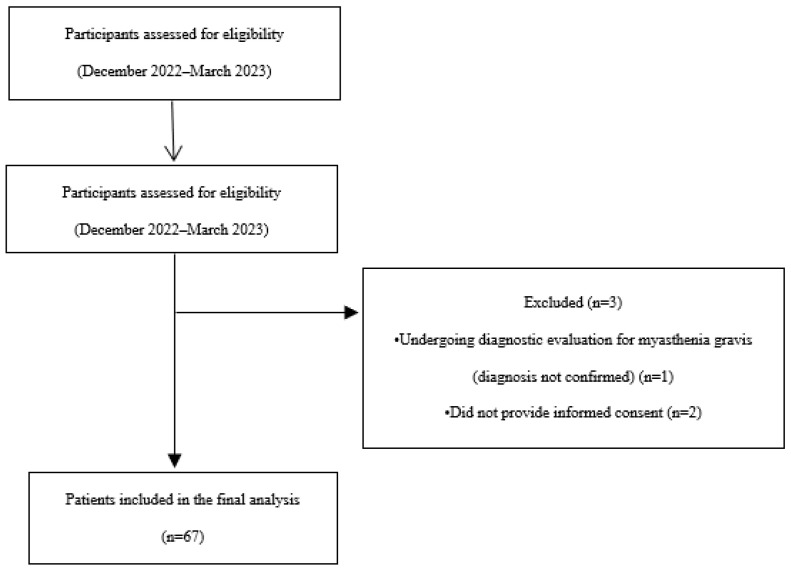
Flowchart of patients selection.

The outpatient department setting was considered appropriate for the purposes of this study, as it allowed access to patients at different stages of myasthenia gravis during routine clinical follow-up, thereby reflecting real-world clinical practice, Figure 1.

### 2.3. Tools

The study used a structured data collection form developed by the authors and six standardized clinical instruments. The author-developed form was designed to collect socio-demographic and clinical information and to obtain patients’ subjective assessments related to their health status and fatigue perception. It was not intended as a psychometric scale but as a descriptive tool supporting the interpretation of standardized questionnaire results.

- The author-developed form consisted of three sections: socio-demographic data, clinical characteristics related to myasthenia gravis (including disease duration, clinical form, and current treatment), and selected single-item questions assessing patients’ subjective perceptions of health, disease impact, and the extent to which fatigue affects daily life and is understood by others. The single-item questions assessing fatigue perception and social understanding used Likert-type response scales and were intended to provide a concise descriptive assessment rather than a psychometric evaluation.
- The author-developed data collection form was based on the assumption that fatigue in myasthenia gravis is not solely a physical symptom but also a subjective experience influenced by psychological and social factors. Therefore, the form was designed to capture selected descriptive variables considered clinically relevant for fatigue perception, including subjective health assessment, perceived impact of fatigue on daily functioning, and perceived understanding of fatigue by others. The wording of the author-developed questionnaire items is provided in Supplementary Material S1.
- The level of perceived fatigue was assessed using the Chalder Fatigue Scale (CFQ), developed by T. Chalder et al. in 1993 as a self-report tool for measuring fatigue severity in the general population and in patients with various medical conditions [11]. The questionnaire consists of 11 questions covering two dimensions: physical fatigue (including lack of energy, muscle weakness, increased need for rest) and mental/cognitive fatigue (including difficulty concentrating, memory problems) [11]. Responses are given on a four-point Likert scale (‘less than usual’, ‘no more than usual’, ‘more than usual’, ‘much more than usual’). Depending on the scoring method used, two methods are applied:
  1. bimodal scoring (0–0–1–1), in which the total score ranges from 0 to 11 points,
  2. Likert scoring (0–1–2–3), which allows for a score ranging from 0 to 33 points [12].

There is no uniform, universal cut-off point in the interpretation of the results, but in the bimodal version, a score of ≥4 points indicates clinically significant fatigue (‘caseness fatigue’) [13]. The Likert version allows for a more detailed assessment of symptom severity and monitoring of changes over time. Higher scores in both systems indicate higher levels of fatigue. All statistical analyses were conducted using the Likert scoring method of the Chalder Fatigue Questionnaire (CFQ).

The scale is characterized by high internal consistency (Cronbach’s α value is usually in the range of 0.86–0.92) and good psychometric validity, both in terms of measuring physical and cognitive fatigue [12]. However, it should be emphasized that a ceiling effect may occur in the population of patients with very severe symptoms, limiting the ability to detect any further deterioration [11]. In the present study, the CFQ demonstrated high internal consistency (Cronbach’s α = 0.90).

- The MG-ADL (Myasthenia Gravis Activity of Daily Living) questionnaire was used to assess the ability to perform daily activities in patients with myasthenia gravis. This tool is used to measure the functional ability of patients in everyday life. It consists of eight items: two related to visual disturbances, three to bulbar functions, one to respiratory functions, and two to limb function. Each item is scored on a scale of 0–3 points, giving a possible total score of 0 to 24. The higher the score, the greater the limitations in daily functioning. The questionnaire is reliable with Cronbach’s Alpha 0.70 [14]. The questionnaire also allows us to identify the areas in which the patient experiences the most or least difficulties [15]. In the present study, the MG-ADL scale showed acceptable internal consistency (Cronbach’s α = 0.70).
- Quality of life was assessed using a shortened 15-point questionnaire, MG-QOL15 (Myasthenia Gravis Quality of Life 15), created specifically for this group of patients. It is an extract from the comprehensive 60-item version of MG-QOL. The MG-QOL15 has demonstrated excellent internal consistency in the original validation work (Cronbach’s α = 0.89). The selected questions were chosen for their accuracy and sensitivity. Responses are given on a five-point Likert scale (0—not at all, 1—a little, 2—somewhat, 3—significantly, 4—very much). The maximum score is 60 points. No clear cut-off points have been specified, but higher scores indicate a poorer quality of life. Studies usually report an average score for the population analyzed [16]. In the present study, the MG-QOL15 demonstrated excellent internal consistency (Cronbach’s α = 0.94).
- A modified version of the HADS-M (Hospital Anxiety and Depression Scale—Modified Version), originally developed by A. Zigmond and R. Snaith and adapted into Polish by M. Majkowicz, K. de Walden-Gałuszko and G. Chojnacka-Szawłowska, was used to assess the severity of anxiety, depression and irritability. The scale includes two independent subscales (anxiety and depression), each consisting of seven statements, and two additional questions about irritability. Responses are given on a four-point Likert scale (0–3 points). The total score in each subscale ranges from 0 to 21, while for questions about irritability it ranges from 0 to 6. Scores of 0–7 are considered normal, 8–10 borderline, and 11–21 indicate significant disturbances [17]. A review of the HADS validation data indicates that the scale performs well as a measure of symptom severity and screening [18]. In the present study, the internal consistency of the HADS subscales was good (Cronbach’s α = 0.84 for anxiety and α = 0.79 for depression).
- Coping with stress was assessed using the Mini-COPE questionnaire, which consists of 28 questions relating to typical reactions in difficult situations. Participants respond on a four-point scale (1—almost never, 2—rarely, 3—often, 4—almost always). This tool, designed to assess both healthy and unhealthy adults, describes 14 coping strategies grouped into four categories: active coping, helplessness, support seeking and avoidance strategies. It also takes into account three separate strategies: turning to religion, acceptance, and sense of humor. This makes it possible to determine which ways of coping with stress are dominant among patients [19]. Due to the two-item structure of individual coping strategies, internal consistency was not assessed using Cronbach’s alpha.
- Adherence to therapeutic recommendations was assessed using the ACDS (Adherence in Chronic Disease Scale), which is used in patients with chronic diseases. The questionnaire consists of seven questions, each with five possible answers. Questions 1–5 refer directly to behaviors related to the implementation of recommendations, while the last two concern factors and beliefs that may influence adherence. The total score ranges from 0 to 28 points. A score below 20 points indicates low adherence, a score of 21–26 points indicates moderate adherence, and a score of ≥27 points indicates high adherence. Only the highest level indicates full adherence to therapeutic recommendations [20]. The ACDS has demonstrated good internal consistency, but in the present study, the internal consistency of the ACDS was low (Cronbach’s α = 0.43); therefore, results involving adherence should be interpreted with caution [21].

Internal consistency indices for all standardized instruments used in the study are reported to support the reliability of the measurements in the present sample. As the author-developed form consisted of single-item descriptive questions, internal consistency or factor structure analyses were not applicable.

### 2.4. Data Collection

Data were collected using an anonymous paper-based questionnaire during outpatient clinic visits. Participants completed the questionnaires independently in the presence of a trained member of the research team, who provided clarification when necessary. Other members of the research team involved in data collection were healthcare professionals with experience in neurology and were trained in standardized data collection procedures prior to study initiation.

### 2.5. Statistical Analysis

Statistical analysis was performed using Pearson’s correlation coefficient to assess the relationship between fatigue and functioning, quality of life, social support, coping strategy and adherence. Values of *p* < 0.05 were considered statistically significant. To account for multiple testing, *p*-values were adjusted using the Benjamini–Hochberg false discovery rate (FDR) procedure. Data distributions were assessed using descriptive statistics and tests of normality. Despite some deviations from normality, Pearson’s correlation coefficient was used due to its robustness in moderate sample sizes and for composite scale scores. Linearity was evaluated through visual inspection of scatterplots and was deemed satisfactory.

All statistical calculations were performed using IBM SPSS Statistics 23 and Microsoft Excel 2016.

Due to the exploratory nature of the study, correlation analyses were performed to examine associations between fatigue perception and clinical and psychosocial variables.

## 3. Results

### 3.1. Characteristics of the Study Group

The study group consisted of 67 people, predominantly women (61.2%), with an average age of 53 (M = 53.40, SD = 14.05, range 18–81). Most respondents (62.7%) completed secondary education (primary education 4.5%, higher education 32.8%), and nearly 40% remained professionally active. Patients lived in rural areas (38.8%) or in cities with fewer than 100,000 inhabitants (20.9%), 100,000–500,000 inhabitants (22.4%), or more than 500,000 inhabitants (17.9%).

Clinically, nearly 75% of the respondents had generalized myasthenia gravis, and 25.4% had ocular myasthenia gravis. The disease was mostly diagnosed over 10 years ago (43.3%), while the smallest group consisted of patients who have had the disease for less than a year (9%). Over half of the patients (64.6%) took cholinesterase inhibitors (e.g., Mestinon, Metylase). The most prevalent clinical manifestations included drooping eyelids (15.7%), weakness of the lower limb muscles (17.6%), weakness of the hand muscles in everyday activities (16.5%) and diplopia (12.3%).

### 3.2. Perceived Fatigue

The studies show that the problem of fatigue is extremely significant in the course of myasthenia gravis. A total of over 70% of patients complain of constant and frequent fatigue. Only 4.5% of the study group report that fatigue is rarely felt, Table 1.

### 3.3. Functioning, Quality of Life and Psychological Factors—Descriptive Results

Functioning, quality of life, psychological factors, and adherence to therapeutic recommendations were assessed using standardized questionnaires.

Daily functioning assessed with the Myasthenia Gravis Activities of Daily Living (MG-ADL) scale showed a mean score of 0.44 ± 0.53. Quality of life measured using the MG-QoL15 questionnaire had a mean score of 1.69 ± 1.22.

Psychological factors assessed with the HADS-M scale showed a mean score of 1.59 ± 0.78. Adherence to therapeutic recommendations measured with the ACDS had a mean score of 3.51 ± 0.70.23.

### 3.4. Functioning of Patients with Myasthenia Gravis (MG-ADL) and Quality of Life (MG-QoL15)

Further analyses using Pearson’s correlation coefficient showed statistically significant associations between subjectively perceived fatigue and the overall assessment of the functioning of patients with myasthenia gravis and their quality of life. The higher the score obtained in the questionnaire assessing the functioning of patients with myasthenia gravis (i.e., the worse the patient’s functioning), the greater the fatigue. Similarly, the higher the score in the questionnaire measuring quality of life (i.e., the worse the quality of life), the greater the patient’s fatigue. The links between perceived fatigue and the extent to which fatigue impairs life were also significant. However, no statistically significant relationship was found between the intensity of fatigue and the extent to which others understand patients with this problem. Overall, the observed correlations were moderate to strong and remained statistically significant after FDR correction, with confidence intervals not including zero, except for the perception of being understood by others. Detailed data are presented in Table 2.

Analysis of the data presented in Table 3 showed that the extent to which fatigue hinders the patient’s daily life was significantly associated with several psychological and disease-related variables, whereas the extent to which fatigue is understood by others was associated with a different pattern of relationships. Specifically, greater fatigue-related interference with daily life was associated with higher levels of depressive symptoms, poorer current health assessment, lower disease acceptance, and a greater perceived impact of the disease on daily life.

In contrast, the extent to which fatigue was perceived as being understood others was significantly negatively associated with anxiety and depressive symptoms, and positively associated with disease acceptance, perceived disease control, and disease understanding.

Overall, the observed associations were of small to moderate magnitude. While several correlations remained statistically significant after FDR correction, the confidence intervals indicated variability in effect size, suggesting that the strength of these relationships may differ across individuals.

### 3.5. Stress Coping Strategies (Mini-COPE)

Analysis of the data presented in Table 4 showed that, after FDR correction, only one coping strategy was significantly associated with the analyzed variables. The extent to which fatigue was perceived as being understood by others was positively associated with acceptance as a way of coping with stress. No statistically significant associations were observed between the extent to which fatigue hinders daily life and any of the coping strategies assessed using the Mini-COPE questionnaire after FDR correction.

## 4. Discussion

### 4.1. Perceived Fatigue in Myasthenia Gravis

The conducted research confirms that fatigue is one of the most burdensome and common symptoms in patients with myasthenia gravis. Over 70% of respondents reported chronic fatigue of a constant or frequent nature, indicating that a high percentage of patients experience this symptom in their daily lives. These findings are consistent with previous studies indicating that fatigue in myasthenia gravis is highly prevalent and associated with functional limitations and reduced quality of life, as well as increased psychosocial burden [5,6]. These findings are consistent with previous studies indicating that fatigue in myasthenia gravis is a highly prevalent and burdensome symptom that significantly coexists with emotional distress, functional limitations, and reduced quality of life [5,6]. However, due to the cross-sectional nature of the study, these relationships should be interpreted as associations rather than causal effects.

### 4.2. Fatigue Versus Functioning and Quality of Life (MG-ADL and MG-QoL15)

Even at the descriptive level, patients with myasthenia gravis in the present study were characterized by moderate limitations in daily functioning and reduced quality of life. This provides an important context for further correlational analyses, indicating that fatigue occurs in individuals who are already significantly burdened by the consequences of the disease.

Statistical analyses showed that subjectively perceived fatigue correlated significantly with both the patients’ level of functioning and their quality of life. The worse the results obtained in the MG-ADL and MG-QoL15 questionnaires, the higher the intensity of fatigue. The observed associations indicate that higher levels of perceived fatigue are related to poorer functioning and lower quality of life in patients with myasthenia gravis, with confidence intervals suggesting moderate to strong relationships. Similar associations have been reported in previous studies, suggesting that fatigue frequently co-occurs with functional impairment and reduced quality of life rather than acting as an isolated symptom [6,22]. Nevertheless, the present findings do not allow conclusions regarding the directionality of these relationships. The strength and consistency of these associations underscore the clinical relevance of fatigue as a key correlate of functional impairment and reduced quality of life in myasthenia gravis.

### 4.3. The Subjective Perception of the Degree to Which Fatigue Interferes with Everyday Functioning

The present findings indicate that the extent to which fatigue interferes with daily life is associated with several psychosocial variables relevant to disease adaptation. Greater perceived interference of fatigue was related to higher levels of depressive symptoms, lower disease acceptance and perceived control, and poorer self-rated health. These associations suggest that fatigue perception may be closely linked to patients’ psychological well-being and subjective experience of living with myasthenia gravis. Importantly, these relationships should be interpreted within a correlational framework, as the study design does not permit causal inference [23].

### 4.4. Understanding of Fatigue by Others—Social Context

An important aspect of this study concerns the perceived understanding of fatigue by others and its psychosocial context. While no significant association was observed between fatigue severity and the extent to which fatigue was understood by others, greater perceived understanding was associated with lower levels of anxiety and depressive symptoms, higher disease acceptance and perceived control. These findings suggest that social understanding of fatigue may be linked to more favorable psychological and behavioral outcomes, even if it is not directly related to fatigue intensity itself. Similar observations have been reported in previous studies emphasizing the role of social support and psychosocial resources in shaping patients’ adaptation to chronic neurological diseases [22,23].

### 4.5. Stress Coping Strategies (Mini-COPE)

The results concerning coping with stress were also noteworthy. After correction for multiple testing, the perception of fatigue being understood by others was associated only with greater use of acceptance as a coping strategy, whereas no statistically significant associations were observed between the extent to which fatigue hinders everyday life and any coping strategies. The literature by Boldingh et al., 2015 emphasizes that adaptive coping strategies, such as acceptance and actively seeking support, can reduce the impact of chronic fatigue and improve patient functioning [22]. The results obtained therefore suggest that therapeutic interventions aimed at supporting constructive coping strategies and strengthening social support may be important in the care of patients with MG [22].

In summary, the presented results indicate that fatigue in myasthenia gravis is not only a somatic symptom but is multidimensional in nature, also encompassing the psychosocial, emotional and cognitive functioning of patients.

Greater fatigue severity was associated with poorer quality of life and higher levels of anxiety and depressive symptoms, as well as less favorable disease-related perceptions. At the same time, perceived understanding of fatigue by others was associated with more favorable psychosocial outcomes, although these relationships were generally small to moderate in magnitude.

Although only one coping strategy was significantly associated with fatigue perception variables, these findings highlight the potential importance of adaptive coping mechanisms (especially acceptance) in the psychosocial functioning of patients with myasthenia gravis. Further studies using longitudinal designs are needed to clarify the role of coping strategies over time and to determine their potential clinical relevance [23].

### 4.6. Limitations

This study has several limitations that should be considered when interpreting the findings. First, the cross-sectional design does not allow for causal inferences regarding the relationships between fatigue perception and psychological, social, or clinical variables. Second, the relatively small sample size and recruitment from a single clinical center may limit the generalizability of the results to the broader population of patients with myasthenia gravis.

Additionally, the use of correlation analyses across multiple variables may increase the risk of type I error. The present analyses should therefore be regarded as exploratory. Future studies with larger samples and longitudinal designs, as well as multivariable analytical approaches, are needed to confirm and further clarify the observed associations.

Furthermore, although standardized and validated instruments were used to assess key clinical and psychosocial variables, some aspects of fatigue perception and social understanding were assessed using single-item measures, which may limit measurement precision. Finally, objective clinical indicators of disease severity and treatment characteristics were not controlled for in the analyses and should be included in future research.

## 5. Conclusions

Fatigue remains a highly prevalent and burdensome symptom in patients with myasthenia gravis, closely associated with impaired daily functioning, reduced quality of life, and psychological distress. The findings of this study highlight that not only fatigue severity but also patients’ subjective perception of how fatigue interferes with everyday life is related to important psychosocial aspects of living with the disease.

Moreover, the perceived understanding of fatigue by others appears to be associated with more favorable psychological and behavioral outcomes, including lower levels of anxiety and depressive symptoms, greater disease acceptance, and better adherence to treatment recommendations. Although these relationships should be interpreted with caution due to the cross-sectional and exploratory nature of the study, they underscore the relevance of psychosocial and social-contextual factors in the experience of fatigue in myasthenia gravis.

These findings support the need for a comprehensive approach to the management of myasthenia gravis that extends beyond pharmacological treatment and incorporates psychological support, patient education, and attention to social understanding and coping resources. Future research using longitudinal designs and larger samples is warranted to further clarify the role of fatigue perception and psychosocial factors in this population.

## Figures and Tables

**Table 1:** Percentage distribution of subjectively perceived fatigue in the study group of patients with myasthenia gravis.

Fatigue (Frequency of Occurrence in Patients)	N	%
I feel fatigued all the time	10	14.9
I feel fatigued often	37	55.2
I feel fatigued occasionally	17	25.4
I rarely feel fatigued	3	4.5

**Table 2:** Correlations between fatigue experienced by patients, functioning and quality of life in myasthenia gravis, and the extent to which fatigue hinders life and is understood by others.

	Fatigue (CFQ) r [CI]
Functioning in myasthenia gravis (MGADL)	0.55 * [0.35, 0.70]
Quality of life in myasthenia gravis (MGQoL)	0.42 * [0.20, 0.60]
The extent to which fatigue hinders life	0.45 * [0.23, 0.62]
The extent to which fatigue is understood by others	−0.03 [−0.26, 0.22]

* pFDR < 0.05 (Benjamini–Hochberg correction); Pearson’s r reported.

**Table 3:** Pearson correlations between the perception of fatigue hindering life, how well others understand the fatigue experienced by the patient, and anxiety, symptoms of depression, current health assessment, perception of myasthenia gravis, and the extent to which the patient adheres to treatment.

	The Extent to Which Fatigue Hinders Life r [CI]	The Extent to Which Fatigue Is Understood by Others r [CI]
Anxiety (HADS-A)	0.27 [0.03, 0.48]	−0.37 * [−0.57, −0.15]
Depression (HADS-D)	0.29 * [0.06, 0.50]	−0.32 * [−0.53, −0.09]
Current health assessment	−0.41 * [−0.60, −0.20]	−0.17 [−0.39, 0.08]
Disease acceptance	−0.31 * [−0.51, −0.08]	0.30 * [0.06–0.50]
Disease control	−0.26 [−0.48, −0.02]	0.29 * [0.05–0.49]
Disease understanding	−0.29 [−0.45, 0.01]	0.31 * [0.08–0.52]
Disease impact	0.36 * [0.14, 0.56]	−0.04 [−0.28, 0.20]
Adherence (ACDS)	−0.02 [0.26, 0.22]	0.25 [0.01–0.47]

* pFDR < 0.05 (Benjamini–Hochberg correction); Pearson’s r reported.

**Table 4:** Pearson’s correlations between the feeling that fatigue hinders life and how well others understand the patient’s fatigue, and ways of coping with stress (Mini Cope).

	The Extent to Which Fatigue Hinders Life	The Extent to Which Fatigue Is Understood by Others
Active coping	−0.17 [−0.39, 0.07]	0.14 [−0.10, −0.37]
Helplessness	0.21 [−0.03, 0.43]	−0.18 [0.40, 0.06]
Seeking support	−0.29 [−0.50, −0.05]	0.20 [−0.04, 0.42]
Religion	0.02 [−0.22, 0.26]	0.15 [−0.09, 0.38]
Acceptance	−0.12 [−0.35, 0.12]	0.31 * [0.08, 0.51]
Humor	−0.12 [−0.35, 0.12]	0.08 [−0.16, 0.31]

* pFDR < 0.05 (Benjamini–Hochberg correction); Pearson’s r reported.

## Data Availability

The datasets obtained and analyzed during the current study are available from the corresponding author upon reasonable request. The data are not publicly available due to ethical restriction, as the dataset contains sensitive personal and health-related information that could potentially compromise participant confidentiality.

## Supplementary Materials

The following supporting information can be downloaded at: https://www.mdpi.com/article/10.3390/healthcare14030342/s1, Supplementary Material S1: Author-developed questionnaire items.
