# Supplementary material for: Perceived Fatigue and Associated Psychological Factors in Patients with Myasthenia Gravis
Source: Healthcare (Basel). 2026 Jan 29;14(3):342. doi: 10.3390/healthcare14030342 (PMC12897320; doi:10.3390/healthcare14030342)
Supplement: Supplementary file 1 [file healthcare-14-00342-s001.zip › healthcare-4087049-supplementary.pdf]

Supplementary Material S1. Author-developed questionnaire items

The following single-item questions were used to assess selected aspects of fatigue perception and disease appraisal. All items were rated on an 11-point numeric scale ranging from 0 to 10, with higher scores indicating a greater intensity of the assessed construct.

1. *"To what extent does your disease and its treatment interfere with your daily life?"*
   (0 = does not interfere at all; 10 = interferes very strongly)

2. *"To what extent do you feel you understand your disease and its treatment process?"*
   (0 = do not understand at all; 10 = understand completely)

3. *"To what extent do you feel that your fatigue is understood by others?"*
   (0 = not understood at all; 10 = fully understood)
